# Why are some species older than others? A large-scale study of vertebrates

**DOI:** 10.1186/s12862-016-0646-8

**Published:** 2016-05-04

**Authors:** Laure Cattin, Johan Schuerch, Nicolas Salamin, Sylvain Dubey

**Affiliations:** Department of Ecology and Evolution, Biophore Building, University of Lausanne, 1015 Lausanne, Switzerland; Swiss Institute of Bioinformatics, Quartier Sorge, 1015 Lausanne, Switzerland

**Keywords:** Species age, Intraspecific diversification, Latitude, Viviparity, Oviparity, Colour polymorphism

## Abstract

**Background:**

Strong variations are observed between and within taxonomic groups in the age of extant species and these differences can clarify factors that render species more vulnerable to extinction. Understanding the factors that influence the resilience of species is thus a key component of evolutionary biology, but it is also of prime importance in a context of climate change and for conservation in general. We explored the effect of extrinsic and intrinsic factors on the timing of the oldest diversification event in over 600 vertebrate species distributed worldwide. We used phylogenetic comparative methods to show that color polymorphism, latitude and reproduction (the latter through its interaction with latitude) affected the timing of the oldest diversification event within a species.

**Results:**

Species from higher latitudes tended to be younger, and colour-polymorphic species were older than monomorphic species. Mode of reproduction was important also, in that the age of oviparous species decreased with latitude, whereas no pattern was apparent for viviparous species. Organisms which have already persisted for a long time may be more likely to deal with future modifications of their environment.

**Conclusions:**

Species that are colour polymorphic, viviparous, and/or live at low latitudes have exhibited resilience to past environmental changes, and hence may be better able to deal with current climate change.

**Electronic supplementary material:**

The online version of this article (doi:10.1186/s12862-016-0646-8) contains supplementary material, which is available to authorized users.

## Background

If we wish to predict the resilience of a species to current challenges, such as those caused by climate change, one important source of information may be the past history of that taxon. A species that has persisted through very long periods – and thus, survived across a wide range of environmental conditions – may be more resilient than a recently evolved taxon whose vulnerability has not yet been tested to the same degree. The age of a species can then be considered as a consequence of resilience, which, in turn, will be determined by specific phenotypic traits. All else being equal then, we might thus expect “older” species to be more resilient. An important question is then what are the factors that affect a species’ age?

Previous research shows that a species’ geographic location is linked to how long it has persisted since being formed (defined as the age of species in [1–3]). On average, terrestrial vertebrate species are older in the Southern than in the Northern Hemisphere (invertebrates, plants, fungi and marine vertebrates have never been considered). The severity of the Last Glacial Maximum was different in these two Hemispheres: extensive ice sheets were almost entirely absent from the Southern Hemisphere, whereas large portions of North America and Eurasia were covered by glaciers [4, 5]. These differences placed fewer constraints on organismal viability, enabling populations to persist over extensive areas in the Southern Hemisphere. As a result, the lack of glaciation reduced the loss of genetic diversity [1, 6]. For similar reasons, the latitudinal distribution of organisms might also impact the age of species. For example, Weir and Schluter [7] found that within New World birds and mammals, the age of species, as well as the divergence between sister species is older towards the tropics.

Intrinsic factors might also influence the resilience of species. In particular, it has been proposed that the reproductive strategies and neonatal behaviour, phenotypic characteristics, as well as the presence of intraspecific phenotypic variations, can have an effect on species ages (e.g. [8–12]). In this respect, recent studies have shown that intraspecific colour polymorphism allows species to exploit different habitat types or have broader distributions because of potential differences in behaviour, thermoregulation capacities, or prey–predator interactions (e.g. [13–15]). In addition, a study strictly focusing on snakes highlighted that the age of colour polymorphic species was older than those of monomorphic taxa [12]. In combination, these results suggest that being colour polymorphic enhances the resilience of species. Concerning the reproduction strategies and precociality of neonates, studies on the evolution of viviparity in reptiles suggest that it is a key innovation compared to oviparity, buffering species against the negative impact of past climate fluctuations or enabling them to better exploit cold areas [10, 16, 17]. However, it may also be a dead-end in some circumstances as the transition to viviparity is often irreversible, meaning that cold adapted viviparous species might be at high risk in a context of global warming [17]. Lastly, species with independent (precocial) neonates would be expected to be more resilient. Under changing conditions, independent neonates can move by themselves to find a better environment. In contrast, dependent (altricial) neonates need to be carried during moves, which burdens the parents and lessens their survival rate.

Any investigation of intrinsic factors associated with species age must also consider the indirect effect of body size. It is well known that body size is correlated with generation time and life history traits, as well as with population density. For example, small-bodied species have been shown to be more resilient than large ones (e.g. [9, 18]). Nonetheless, this factor has been neglected in previous studies looking at the age of species.

In the present study, we aim to understand how extrinsic and intrinsic factors influence the resilience of vertebrate taxa and hence their age. Based on a dataset of 600 species worldwide and using phylogenetic comparative methods, we tested for an effect of the geographic location, body size, reproduction mode (oviparity versus viviparity), newborn dependence behaviour (precocial, altricial), and presence of intraspecific colour polymorphism on the timing of the oldest diversification event within species. Our study differs from previous studies by including taxa that are distributed in all areas of the world and we consider in our model all the factors that have been hypothesized to affect the age of species. It is also the first to consider all classes of vertebrates simultaneously and consider for the first time the phylogenetic effects due to shared ancestry between species. It therefore provides a much more complete and clear picture of the factors shaping the resilience of species.

## Results

**Fig. 1:**
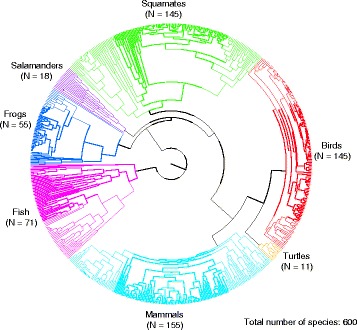
Best tree obtained from MrBayes and used for the comparative analyses

**Fig. 2:**
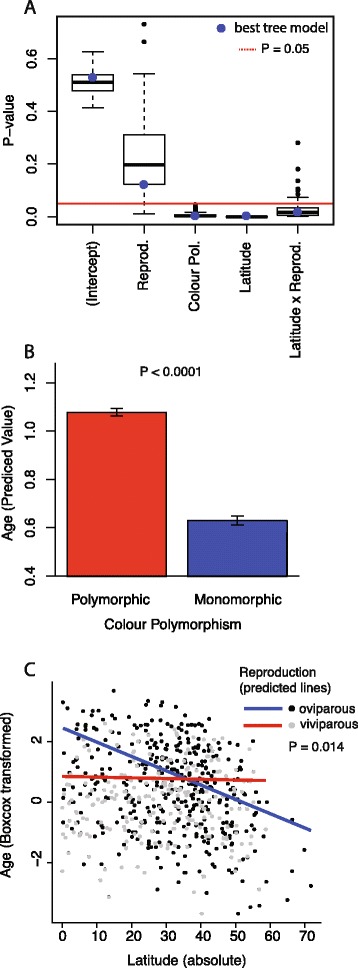
**a** Distribution of *p*-values for the different explanatory factors retained in our final model with the age of species as response variable, for 100 trees sampled from the posterior distribution of trees obtained from MrBayes (best tree: blue dot); Relationships between the age of species and (**b**) the presence or absence of intraspecific colour polymorphism and (**c**) the interaction between the mean latitudinal distribution of species and their reproduction mode (viviparous versus oviparous)

The phylogenetic tree for the 601 vertebrate species (including the shark species *Squalus acanthias* as an outgroup) was congruent with existing knowledge on the relationships of the taxonomic groups included (Fig. 1). Most of the nodes were well supported and the 100 trees sampled from the posterior distribution of trees that were used to account for phylogenetic uncertainty showed small differences (mainly occurring in nodes close to the tips of the tree). The divergence times obtained by maximum likelihood are within the 95 % interval of the posterior samples obtained with BEAST. The two analyses resulted in very similar divergence times and we therefore use the dates obtained by maximum likelihood for the remaining of the analyses. The dates obtained for the main lineages of vertebrates are also congruent with recent large scale phylogenetic reconstructions [19–22]. We used phylogenetic GLS to test the factors affecting the age of species, which was used as the response variable in our analyses. The backward model selection retained three single factors and one interaction in the final phylogenetic GLS model (Fig. 2a; Additional file 1). Colour polymorphism and latitude were highly significant (*p*-value < 0.001; Fig. 2a), while mode of reproduction was maintained due to its significant interaction with latitude. Polymorphic species had a much higher resilience than monomorphic ones (fig. 2b) and, on average, the age of the polymorphic species was (when transformed back into the original scale) 1.86 Myr older than the age of monomorphic ones (Fig. 2b; Additional file 2). Latitude was also significant, but its effect was influenced by the mode of reproduction. Oviparous species showed a strong latitudinal trend, with species living far from the Equator being younger than species living in tropical climates (Fig. 2c). In contrast, viviparous species did not show any latitudinal trend in species age (Fig. 2c).

## Discussion

Our study, based on a dataset of 600 vertebrate species, allowed us to identify several extrinsic and intrinsic factors that influence significantly the age of vertebrate species. It also clarified results from previous studies that were not based on comparative methods, focused on particular taxa or considered fewer factors (see e.g. [1–3, 7, 12]). Overall, absolute mean latitude of the species distribution was negatively correlated with species age and colour-polymorphic species were older than monomorphic species. Moreover, the age of oviparous species decreased with latitude, whereas no such pattern was apparent for viviparous ones.

The latitudinal gradient found in this study is consistent with the results of Weir and Schluter [7], who found that within New World birds and mammals, the age of species, as well as the divergence between sister species, is younger towards the poles. Using interspecific divergences, they estimated that the highest recent speciation and extinction rates occurred at high latitudes and declined toward the tropics. This pattern is likely explained by the presence of more stable climatic conditions in the tropics than in temperate areas during Pleistocene climatic fluctuations [5]. According to Weir and Schluter [7], this climatic stability leads to a lower species turnover in the tropics compared to temperate areas, resulting in a lower diversity in the latter. Based on the similarity between our results and those of Weir and Schluter [7] in term of intraspecific latitudinal gradient, this pattern might be generally valid for vertebrate species from both Hemispheres. However, the situation is likely more complex. Indeed, the age of viviparous vertebrate species was not influenced by their mean latitudinal distribution, whereas the age of oviparous species decreased towards the poles. This result suggests a higher resilience of viviparous species to cold climate, and can be attributed to their buffering capacity against inadequate environmental conditions during the embryogenesis. This is particularly true for heliothermic vertebrates, for which a cold year might result in an absence of reproduction if favourable nesting sites are absent. In contrast, viviparous species might be able to better deal with poor environmental conditions by modifying their sun-basking activities (for a review see e.g. [10, 16, 23]). For instance, Pincheira-Donoso et al., [17], found that, in a widespread South American lizard genus (*Liolaemus*), the evolution of viviparity was associated with a radiation into cold climates. This argument is not relevant to birds or placental and marsupial mammals (as opposed to egg-laying monotremes) because their breeding type is constant and they are more widely distributed. However, the argument can be extended to other ectothermic vertebrates (fish and amphibians).

Body colour plays a major role in the evolution of organisms as it is involved, for instance, in thermoregulation, prey–predator interaction, behaviour, protection against UV-light or abrasive agents [24]. Therefore, the presence of intraspecific colour polymorphism may promote the ecological success of species as previously shown for squamates [14, 15], amphibians [25] and owls [26], through their capacity to exploit a larger range of habitat types. In turn, polymorphic species might be more resilient to past and future climatic fluctuations. Our results are also in agreement with the study of Pizzatto & Dubey [12], which found that colour-polymorphic snake species were older than monomorphic species.

Recent studies focusing on temperate vertebrate taxa highlighted that species from the southern Hemisphere are older than those from the northern, and that differences in past climate are likely responsible for this pattern [1–3]. Interestingly, the Hemisphere of origin is not impacting the age of species in our study, which is likely due to the inclusion of additional intrinsic and extrinsic factors and to the use of phylogenetic comparative methods. Indeed, when the Hemisphere of origin is included in our analyses without accounting for the phylogenetic relationships between taxa, southern species remained significantly older than northern ones (see Additional file 3). The same is true when strictly temperate species of our dataset are included (the use of a comparative method leading to a marginally significant result; Additional file 3). These differences illustrate the importance of considering an array of factors in such analyses. In addition, the phylogenetic relationships of studied taxa might deeply influence such studies, keeping in mind that northern and southern Hemisphere temperate fauna are distantly related genetically, numerous families and genera being solely distributed in one of the two Hemispheres.

## Conclusions

In conclusion, our findings suggest that intrinsic and extrinsic variables might contribute to the resilience of species to past environmental changes and hence are relevant in a context of climate change. Overall and independently of current threats, organisms which exhibit ancient intraspecific diversification events are more likely to deal with future modifications of their environment, those species being colour polymorphic, viviparous, or living at low latitudes.

## Methods

### Species sampling and traits

We gathered published phylogenetic and phylogeographic studies for vertebrate species using searches within Scopus and ISI Web of Knowledge. We defined the age of a species (in Myr) as the oldest intraspecific diversification event, which is an estimate of the amount of time since extant populations last shared a common ancestor [1–3, 7]. Species ages were obtained directly from the studies included in our dataset and hence were not estimated in the present study. In addition, only studies including a reasonable number of individuals and covering most of the distribution of the species have been included. We excluded non-monophyletic species and used the Catalogue of Life [27] to eliminate synonyms and ambiguities. Our final sampling contained species from 71 freshwater fish, 73 amphibians, 156 reptiles, 145 birds and 155 mammals (See Fig. 1 & Additional file 4).

We collected for each species their geographical distributions (Additional file 4), as represented by the minimum and maximum latitude taken from IUCN distributional polygon data. We calculated the mean latitude (in absolute values) and latitudinal range with ArcGIS® (ESRI 2006). We further categorized the mean latitude of each species to allocate them to Northern (between 10° and 90° N) or Southern (between 10° and 90° S) Hemispheres. Tropical species (between 10°N and 10°S) as well as species found on both Hemispheres were defined as intermediates. Latitude is considered here as a covariate that can represent several aspects of the life-history that cannot be easily associated with the more than 600 species sampled in our study.

We also collected data on life-history traits from published taxonomies and available databases. As our measure of mean adult size, we used snout-vent length for amphibian, reptile and mammal species, common size for fish species and length for bird species. Both sexes were pooled together and averaged. We also recorded the breeding type for each species and we combined ovoviviparous and viviparous species. The mean litter or clutch size was defined as the mean number of offspring for one female and we only considered the number of offspring in a single clutch/litter when multiple birthing took place in one year. We considered as well the behaviour of newborns and modified the scale of Starck & Ricklefs [28] into four levels to account for the larger taxonomic breadth of our study (Precocial - neonates eat by themselves and move freely; semi-precocial - neonates move freely by themselves but rely on the parents for food; semi-altricial - neonates unable to move freely by themselves, but still have the capacity to cling or act to survive; altricial - neonates are fully dependant on their parents). Finally, we defined a species as colour-polymorphic when it exhibited at least two different colour morphs within the same sex and age class. Thus, at least one of the sexes had to be polymorphic for the species to be considered polymorphic. No distinction was made between geographical colour variation and within-population colour-polymorphism. See Additional file 5 for factors retained in our final analyses.

### Phylogenetic analysis

The phylogenetic tree for the 601 species sampled was based on four mitochondrial genes (cytochrome b, COI, ND2, and ND4) and one nuclear gene (Rag1), but not all species in the dataset were represented for the five genes (TreeBase accession number). The shark *Squalus acanthias* was used as an outgroup. The sequences were downloaded from GenBank and aligned using MUSCLE [29]. The best model for each gene defined using Akaike Information Criterion (AIC) as implemented in the phymltest function from the R package APE [30]. We inferred the Maximum Likelihood tree using the BEST tree search algorithm as implemented in PhyML 2.3 [31] and we performed 100 bootstrap replicates. We also inferred a Bayesian phylogenetic tree using MrBayes 3.2 [32] and we allowed the model of substitution to take different parameter values for each gene present in the concatenated dataset. The MCMC was run for 50 millions generations and we sampled trees and parameter values every 1000 generations. We repeated the analyses twice to assess the convergence of the chain.

We estimated divergence times using 11 fossil calibration points obtained from the literature (Additional file 6). Given the size of the phylogenetic tree, a full estimation of the tree topology and the divergence times was not practical. We therefore decided to first use a Maximum Likelihood implementation of a relaxed molecular clock [33] as implemented in the function chronos from the R package APE [30]. We also sampled 100 trees from the posterior distribution of trees obtained by MrBayes and repeated the divergence time analysis to obtain a set of calibrated trees. Second, we used BEAST v. 1.8 [34] to estimate the divergence times using a log-normal relaxed molecular clock while fixing the tree topology to the one obtained by MrBayes. We applied the 11 fossil calibration points using log-normal priors. The means and standard deviations of these prior distributions were selected to include the fossil uncertainties in the 95 % of their density. We used a yule process for the prior on divergence times and ran the MCMC for 10 million generations. We assessed the convergence of the chain using Tracer v. 1.7.5 by looking at the trace of the log-likelihood and the ESS values for each parameter.

### Comparative analysis

We used general least squares (GLS) to understand the effects of intrinsic and extrinsic factors on the age of species. We modified species ages with a Box-Cox transformation (lambda = 0.1) and we included the average latitude (in absolute value), Hemisphere, reproduction mode, newborn behavior, body size (Box-Cox transformed, lambda = −0.3) and the presence of colour polymorphism as covariables in the GLS analysis. We started with a full model that included the single factors and all pairwise interactions. We corrected the correlation structure of the GLS by considering the evolutionary relationships between species and we assumed a Brownian correlation structure based on the phylogenetic trees (lambda parameter = 1). We performed all analyses using the gls function from the R package NLME [35] and used backward model selection to test the factors. Model validation was performed using graphical tools (as proposed in [36]). We finally tested the effect of phylogenic tree construction on the best model output by repeating the GLS analysis 100 times sampling each time a new phylogenetic tree randomly from their posterior distribution.

## Additional files

Additional file 1: Final phylogenetic GLS model. (DOCX 13 kb) Additional file 2: Age variation within factors. (DOCX 13 kb) Additional file 3: The effect of hemisphere on age of species, considering or not the phylogenic correction or/and species from the tropics (below 23° latitude in absolute value). (DOCX 18 kb) Additional file 4: Taxa included in our final dataset. (XLSX 72 kb) Additional file 5: Correlation between intrinsic and extrinsic fatcors relative to the species sample. Covariables retained in the model are represented in bold. (DOCX 15 kb) Additional file 6: Calibration points used in the phylogenetic analyses. (DOCX 23 kb)
