# Supplementary material for: Why are some species older than others? A large-scale study of vertebrates
Source: BMC Evol Biol. 2016 May 4;16:90. doi: 10.1186/s12862-016-0646-8 (PMC4855795; doi:10.1186/s12862-016-0646-8)
Supplement: Additional file 1: — Final phylogenetic GLS model. (DOCX 13 kb) [file 12862_2016_646_MOESM1_ESM.docx]

Additional file 1

| **Variable** | **estimate** | **SE** | **F.value** | **p.value** |
| --- | --- | --- | --- | --- |
| (Intercept) | 1.440 | 2.268 | 0.403 | 0.526 |
| Latitude mean (absolute value) | -0.025 | 0.004 | 39.220 | 0.000 |
| Reproductive mode[viviparous] | -0.804 | 0.515 | 2.440 | 0.119 |
| Colour polymorphism [polymorph] | 0.460 | 0.109 | 17.774 | 0.000 |
| Latitude mean (absolute value) x reproductive mode [viviparous] | 0.023 | 0.009 | 6.040 | 0.014 |
