# Supplementary material for: Why are some species older than others? A large-scale study of vertebrates
Source: BMC Evol Biol. 2016 May 4;16:90. doi: 10.1186/s12862-016-0646-8 (PMC4855795; doi:10.1186/s12862-016-0646-8)
Supplement: Additional file 2: — Age variation within factors. (DOCX 13 kb) [file 12862_2016_646_MOESM2_ESM.docx]

Additional file 2

| **Factors** | **Average ± SE (Myr)** |
| --- | --- |
| **Hemisphere** |  |
| North | 2.337 ± 0.137 |
| South | 3.126 ± 0.301 |
| **Reproductive mode** |  |
| Oviparous | 3.071 ± 0.192 |
| Viviparous | 1.729 ± 0.13 |
| **Newborn Behaviour** |  |
| Altricial | 1.133 ± 0.086 |
| Precocial | 3.57 ± 0.2 |
| **Colour polymorphism** |  |
| Monomorphic | 1.838 ± 0.162 |
| Polymorphic | 3.398 ± 0.205 |
