# Supplementary material for: Why are some species older than others? A large-scale study of vertebrates
Source: BMC Evol Biol. 2016 May 4;16:90. doi: 10.1186/s12862-016-0646-8 (PMC4855795; doi:10.1186/s12862-016-0646-8)
Supplement: Additional file 3: — The effect of hemisphere on age of species, considering or not the phylogenic correction or/and species from the tropics (below 23° latitude in absolute value). (DOCX 18 kb) [file 12862_2016_646_MOESM3_ESM.docx]

**Additional file 3**

The effect of hemisphere on age of species, considering or not the pylogenic correction or/and species from the tropics (below 23° latitude in absolute value)

| **1. With phylogenic correction** |  |  |  |  |  |
| --- | --- | --- | --- | --- | --- |
| *a. all species* |  |  |  |  |  |
|  | df | Estimate | SE | F | *P* |
| Intercept | 1/598 | 0.826 | 2.359 | 0.123 | 0.726 |
| Hemisphere | 1/598 | 0.151 | 0.164 | 0.842 | 0.359 |
|  |  |  |  |  |  |
| *b. without species from tropics* |  |  |  |  |  |
|  | df | Estimate | SE | F | *P* |
| Intercept | 1/413 | 0.777 | 2.212 | 0.124 | 0.725 |
| Hemisphere | 1/413 | -0.413 | 0.241 | 2.950 | 0.087 |
|  |  |  |  |  |  |
| **2. Without phylogenic correction** |  |  |  |  |  |
| *a. all species* |  |  |  |  |  |
|  | df | Estimate | SE | F | *P* |
| Intercept | 1/598 | 0.174 | 0.068 | 6.458 | 0.011 |
| Hemisphere | 1/598 | 0.301 | 0.123 | 5.964 | 0.015 |
|  |  |  |  |  |  |
| *b. without species from tropics* |  |  |  |  |  |
|  | df | Estimate | SE | F | *P* |
| Intercept | 1/413 | 0.107 | 0.079 | 1.851 | 0.174 |
| Hemisphere | 1/413 | 0.407 | 0.160 | 6.442 | 0.012 |
