# Supplementary material for: Why are some species older than others? A large-scale study of vertebrates
Source: BMC Evol Biol. 2016 May 4;16:90. doi: 10.1186/s12862-016-0646-8 (PMC4855795; doi:10.1186/s12862-016-0646-8)
Supplement: Additional file 5: — Correlation between intrinsic and extrinsic fatcors relative to the species sample. Covariables retained in the model are represented in bold. (DOCX 15 kb) [file 12862_2016_646_MOESM5_ESM.docx]

**Additional file 5**

Correlation between intrinsic and extrinsic fatcors relative to the species sample. Covariables retained in the model are represented in bold

|  | **Latitude mean (absolute value)** | Latitude Range | **Hemisphere** | Litter Size | **Reprod. mode** | Thermal strategy | **Newborn Behaviour** | Adult mean size (box-cox tranformed) | **Colour polym.** |
| --- | --- | --- | --- | --- | --- | --- | --- | --- | --- |
| **Latitude mean (absolute value)** | - |  |  |  |  |  |  |  |  |
| Latitude Range | 0.01 | - |  |  |  |  |  |  |  |
| **Hemisphere** | -0.32 | -0.04 | - |  |  |  |  |  |  |
| Litter Size | 0.07 | 0.03 | 0.00 | - |  |  |  |  |  |
| **reproductive mode** | -0.09 | 0.00 | 0.04 | -0.09 | - |  |  |  |  |
| Thermal strategy | -0.05 | 0.46 | 0.04 | -0.11 | 0.31 | - |  |  |  |
| **Newborn Behaviour** | 0.09 | -0.39 | -0.05 | 0.09 | -0.28 | -0.83 | - |  |  |
| Adult mean size (box-cox tranformed) | -0.03 | 0.27 | -0.07 | 0.07 | 0.23 | 0.30 | -0.09 | - |  |
| **Colour polymorphism** | 0.06 | -0.16 | -0.04 | 0.07 | 0.08 | -0.45 | 0.35 | -0.13 | - |
