# Supplementary material for: Why are some species older than others? A large-scale study of vertebrates
Source: BMC Evol Biol. 2016 May 4;16:90. doi: 10.1186/s12862-016-0646-8 (PMC4855795; doi:10.1186/s12862-016-0646-8)
Supplement: Additional file 6: — Calibration points used in the phylogenetic analyses. (DOCX 23 kb) [file 12862_2016_646_MOESM6_ESM.docx]

**Additional file 6**

**Supplementary online materials 1**

Calibration points used in the phylogenetic analyses.

| **Reference** | **Date (Myr)** | **CI (Myr)** | **Taxa** |
| --- | --- | --- | --- |
| Wiens et al. 2006 | 99 | 0.05 | Heloderma – Elgaria |
| Wiens et al. 2006 | 33.7 | 0.05 | Viperidae – Colubridae |
| Benton et al. 2007 | 11.7 | 0.035 | *Rattus* – *Mus* |
| Benton et al. 2007 | 52 | 0.13 | Caniformia – Feliformia |
| Benton et al. 2007 | 23 | 0.13 | Bovinae – Antilopinae |
| Benton et al. 2007 | 51 | 0.03 | Ruminantia – Suiformes |
| Benton et al. 2007 | 76 | 0.08 | Galloanserae – Neoaves |
| Benton et al. 2007 | 340 | 0.018 | Reptiliomorpha – Batrachomorpha |
| Luo et al. 2011 | 160 | 0.065 | Placental – Marsupial |

Benton, M.J.,Donoghue, C.J. (2007). Paleontological evidence to date the tree of life. Mol. Biol. Evol. 24, 26-53.

Luo ZX, Yuan CX, Meng QJ, Q Ji (2011) A Jurassic eutherian mammal and divergence of marsupials and placentals. Nature 476, 442-445.

Wiens, J.J., Brandley, M.C., Reeder, T.W. (2006). Why does a trait evolve multiple times within a clade? Repeated evolution of snakelike body form in squamate reptiles. Evolution 60(1), 123-141.

**Supplementary online materials 2**

Correlation between intrinsic and extrinsic factors relative to the species sample. Factors retained in the model are represented in bold

|  | **Latitude mean (absolute value)** | Latitude Range | **Hemisphere** | Litter Size | **Reprod. mode** | Thermal strategy | **Newborn Behaviour** | Adult mean size (box-cox tranformed) | **Colour polym.** |
| --- | --- | --- | --- | --- | --- | --- | --- | --- | --- |
| **Latitude mean (absolute value)** | - |  |  |  |  |  |  |  |  |
| Latitude Range | 0.01 | - |  |  |  |  |  |  |  |
| **Hemisphere** | -0.32 | -0.04 | - |  |  |  |  |  |  |
| Litter Size | 0.07 | 0.03 | 0.00 | - |  |  |  |  |  |
| **reproductive mode** | -0.09 | 0.00 | 0.04 | -0.09 | - |  |  |  |  |
| Thermal strategy | -0.05 | 0.46 | 0.04 | -0.11 | 0.31 | - |  |  |  |
| **Newborn Behaviour** | 0.09 | -0.39 | -0.05 | 0.09 | -0.28 | -0.83 | - |  |  |
| Adult mean size (box-cox tranformed) | -0.03 | 0.27 | -0.07 | 0.07 | 0.23 | 0.30 | -0.09 | - |  |
| **Colour polymorphism** | 0.06 | -0.16 | -0.04 | 0.07 | 0.08 | -0.45 | 0.35 | -0.13 | - |

**Supplementary online materials 3**

The effect of hemisphere on age of species, considering or not the phylogenic correction or/and species from the tropics (below 23° latitude in absolute value)

| **1. With phylogenic correction** |  |  |  |  |  |
| --- | --- | --- | --- | --- | --- |
| *a. all species* |  |  |  |  |  |
|  | df | Estimate | SE | F | *P* |
| Intercept | 1/598 | 0.826 | 2.359 | 0.123 | 0.726 |
| Hemisphere | 1/598 | 0.151 | 0.164 | 0.842 | 0.359 |
|  |  |  |  |  |  |
| *b. without species from tropics* |  |  |  |  |  |
|  | df | Estimate | SE | F | *P* |
| Intercept | 1/413 | 0.777 | 2.212 | 0.124 | 0.725 |
| Hemisphere | 1/413 | -0.413 | 0.241 | 2.950 | 0.087 |
|  |  |  |  |  |  |
| **2. Without phylogenic correction** |  |  |  |  |  |
| *a. all species* |  |  |  |  |  |
|  | df | Estimate | SE | F | *P* |
| Intercept | 1/598 | 0.174 | 0.068 | 6.458 | 0.011 |
| Hemisphere | 1/598 | 0.301 | 0.123 | 5.964 | 0.015 |
|  |  |  |  |  |  |
| *b. without species from tropics* |  |  |  |  |  |
|  | df | Estimate | SE | F | *P* |
| Intercept | 1/413 | 0.107 | 0.079 | 1.851 | 0.174 |
| Hemisphere | 1/413 | 0.407 | 0.160 | 6.442 | 0.012 |
